# Predictors for Half-Year Outcome of Impairment in Daily Life for Back Pain Patients Referred for Physiotherapy: A Prospective Observational Study

**DOI:** 10.1371/journal.pone.0061587

**Published:** 2013-04-19

**Authors:** Sven Karstens, Katja Hermann, Ingo Froböse, Stephan W. Weiler

**Affiliations:** 1 Institute of Health Promotion and Clinical Movement Science, German Sport University Cologne, Cologne, Germany; 2 Department of General Practice and Health Services Research, University Hospital Heidelberg, Heidelberg, Germany; 3 Audi Medical Services, Audi, Ingolstadt, Germany; 4 Department of Occupational Medicine, University Medical Center Schleswig-Holstein, Campus Luebeck, Luebeck, Germany; University of South Australia, Australia

## Abstract

**Background and Objective:**

From observational studies, there is only sparse information available on the predictors of development of impairment in daily life for patients receiving physiotherapy. Therefore, our aim was to identify factors which predict impairment in daily life for patients with back pain 6 months after receiving physiotherapy.

**Methods:**

We conducted a prospective cohort study with 6-month follow-up. Patients were enrolled for treatment in private physiotherapy practices. Patients with a first physiotherapy referral because of thoracic or low back pain, aged 18 to 65 years were included. Primary outcome impairment was measured utilising the 16-item version of the Musculoskeletal Function Assessment Questionnaire. Therapy was documented on a standardized form. Baseline scores for impairment in daily life, symptom characteristics, sociodemographic and psychosocial factors, physical activity, nicotine consumption, intake of analgesics, comorbidity and delivered primary therapy approach were investigated as possible predictors. Univariate and multiple linear regression analyses were performed.

**Results:**

A total of 792 patients participated in the study (59% female, mean age 44.4 (SD 11.4), with 6-month follow-up results available from 391 patients. In univariate analysis 17 variables reached significance. In multiple linear regression identified predictors were: impairment in daily life before therapy, mental disorders, duration of the complaints, self-prognosis on work ability, rheumatoid arthritis, age, form of stress at work and physical activity. The variables explain 34% of variance (adjusted R^2^, p<0.001).

**Conclusions:**

With minimal information available from observational studies on the predictors of development of back problems for physiotherapy patients, this study adds new knowledge for forming appropriate referral guidelines. Impairment in daily life before therapy, mental disorder as comorbidity and the duration of the complaints can be named as outstanding factors. The results of this study can be used to facilitate comparison of patient therapy goals with the prognosis in everyday practice.

## Introduction

Back pain frequently leads to a limitation in quality of life and working ability [1]. Patients with chronic complaints may suffer from distinct restrictions in their social life [2], [3]. Sets of factors useful for prediction of the transition from acute to chronic status include both biomedical and psychosocial aspects [4]. Physiotherapy referrals for treatment are often made, and therapists can positively influence the various factors [5], [6], [7]. On average, in 2008, every seventh person insured with a major German health insurance company received physical therapy [8]. In about 40 percent of cases, the diagnoses related to low back pain [9]. Commonly in therapy utilized approaches include exercise as active and manual therapy, or physical strategies like electrotherapy as passive approaches. Effectivity for these approaches differs: largest effect sizes were shown for exercise, which reaches a level comparable to acupuncture or behavioral therapy [10].

Various studies have been performed to investigate whether referrals for rehabilitation have been appropriate. In this context, Jensen et al. criticize decision-making on the need for rehabilitation as generally being based on expert opinion and thus being non-transparent [11]. They demonstrated that neither physicians among themselves nor physicians and physiotherapists come to corresponding results. Similar results are provided by Archer et al. and Wagemakers et al. [12], [13]. Important information for the referral process is the therapy prognosis. Consequently, models to determine whether the issued referrals have been adequate, regard this factor as essential [14]. However, determining the prognosis is particularly difficult in the case of low back pain patients. Whilst there is extensive information on the natural course of the complaints, only sparse information is available from observational studies on the predictors of development of impairment in daily life specifically under physiotherapy [4], [15]. In a recently published systematic review, Verkerk et al. identified some relevant studies. They have shown that for back pain patients different predictors for impairment in daily life exist, but only in a few studies interactions between predictors and physiotherapy were examined and the predictors were mainly investigated only once [16]. Exceptions in which conservative approaches were taken are those conducted by Underwood et al. and Bekkering et al. [17], [18]. An additional value of these large trials (n≥500) is the setting, since patient samples were from primary health care, which occurs rather seldom. Harms et al. accomplished a cohort study at a multidisciplinary back pain clinic, in which among others physiotherapists were practicing [19].

For patients undergoing spine surgery, Mannion and Elfering give an overview for predictors [20], but therapy-related predictors are only poorly considered and in the private practice sector these patients are only a minority of the patients.

The objective of this study was to identify factors which predict impairment in daily life for patients with back pain 6 months after receiving physiotherapy.

## Methods

Data were collected in a prospective, multicenter cohort study with six-month catamnesis. Patients with thoracic or low-back pain related diagnosis that were referred for physiotherapy by a physician were consecutively admitted to the physiotherapy-centres under consideration of the inclusion and exclusion criteria depicted in **Table 1**. Assignment was based on the standardised referral code “WS” meaning back, as marked on the corresponding form by the physician [21]. The criterion specific back pain meant that patients with serious traumatic conditions or inflammatory rheumatic diseases as referral diagnosis were excluded. Patients with nerve root irritation were included.

**Table 1:** Inclusion and exclusion criteria.

**Inclusion criteria**	Thoracic- or low-back-related diagnosis
	First prescription* of physical therapy (according to form)
	Age between 18 and 65
**Exclusion criteria**	Specific back pain (e.g. Bechterew disease or fracture)
	Not capable of reading, writing and/or understanding German language
	Prescription for massage or lymph drainage as primary therapy

* “First prescription” according to German regulations means no therapy for at least 12 weeks.

Outcomes were measured using the German 16-item-version of the Musculoskeletal Function Assessment Questionnaire [22]. The well known 46-item instrument (SMFA) has been implemented in many countries around the world [23], [24]. The questionnaire comprises two subscales with the underlying constructs impairment (BI) and dysfunction (FI). The instrument is scaled from 0–100 with 0 signifying no and 100 maximal limitation. The result is indicated as “percent”-value. Wollmerstedt et al. have shown good psychometric properties for the questionnaire in various patient groups. Internal consistency for functioning is α≥0.86 and α≥0.78 for impairment respectively. Construct validity was determined by correlation with the corresponding SMFA-subscales, resulting in r≥0.93 for the FI and r≥0.87 for the BI [22], [24].

Potential prognostic factors comprised sociodemographics, diagnosis and symptoms, behavioural aspects, comorbidities and psychosocial factors.

All independent variables including coding are presented in **Table 2**. Sociodemographics were assessed referring to standards set by an epidemiological expert panel [25]. The diagnostic subgroups were determined in a qualitative assessment, using information given on the referral form. Examples for diagnosis assigned to the different groups are depicted in **Table 3**. Pain intensity was measured using an 11-point box scale [26]. Nicotine consumption and physical activity were assessed using self-developed questions. Comorbidities and psychosocial factors were assessed using the Work Ability Index Questionnaire (WAI) [27], [28]. Comorbidities were identified with the WAI sickness list. Mental resources were determined via a subscale of the WAI, which encompasses 3 questions concerning enjoyment of regular daily activities, being active and alert and being hopeful about the future. Self-prognosis on work ability was assessed through a single item WAI-dimension.

**Table 2:** Independent variables.

Independent Variables		Characteristic/value label
Sociodemographic details	Age	scale
	Gender	1 = female, 2 = male
Diagnosis and Symptoms	Subgroup nonspecific, subgroup thoracic, subgroup disc/root irritation, post surgery	1 = yes, 0 = no
	Impairment/pain intensity prior to treatment	scale
	Duration of complaints ≥½ year, radiation into lower extremity, multifocal complaints	1 = yes, 0 = no
Behavioural factors	Physically active, smoker, intake of analgesics	1 = yes, 0 = no
	Body mass index,	scale
Comorbidities	Rheumatoid arthritis, mental disorder, neurological-sensory disease, genitourinary or digestive disease, tumours, diabetes	1 = yes, 0 = no
Psychosocial Factors	Employed, white collar worker, good self-prognosis on work ability in 2 years	1 = yes, 0 = no
	Mental resources	scale
Primary therapy	Active, passive	1 = yes, 0 = no

**Table 3:** Diagnostic Subgroups; examples for assigned referral-diagnosis.

Subgroup	Examples for assigned referral-diagnosis
- nonspecific	Back pain, low back pain, sacroiliac joint pain, sacroiliac joint dysfunction, lumbar spine blockage
- disc/root irritation	Lumbar intervertebral disc displacement, radiculopathy segment L4, lumbosacral disc displacement, slipped disc L3
- thoracic	thoracic spine pain, thoracic spine blockage

Treatment was not influenced by the investigators. It was documented on a standardized form by the therapists after the final session. They had to select from the options depicted in **Table 4**. One option was to mark as primary approach, as many as useful as secondary approaches.

**Table 4:** Documentation categories therapy.

1. Therapeutic exercises	7. Manual therapy	13. Ultrasound
2. Stretching exercises	8. Traction	14. Patient education
3. Proprioception	9. Massage	15. Active assisted exercises
4. Strength training (including machines)	10. Cold therapy	16. Other therapy (free text)
5. Home exercises	11. Heat therapy	
6. Activities of daily life	12. Electrotherapy	

The patients were enrolled between May 2007 and August 2008. Questioning took place immediately before the first therapy session (t1) and 6 months after treatment (t2). The latter was accomplished via mail.

Ethical approval was granted by the Ethics Committee of the University of Luebeck, Germany (registration ID: 07-019). All patients gave their written informed consent for participation, before entering the study in the participating practice.

### Statistics

A multiple linear regression model was calculated [29]. As dependent variable the impairment subscale of the Musculoskeletal Function Assessment Questionnaire 6 months after therapy was set [22]. Before the selection procedure, the independent variables of each case were checked for extremes. Cases were eliminated as outliers, if their value exceeded or presented a shortfall of the arithmetic average by 3 standard deviations. To enable inclusion of treatment into the analyses, the primary approaches were assigned to two variables: active (see **Table 4**, treatment 1–6) and passive treatment (treatment 7–13). Patient education, active assisted exercises and “other therapy” were not ascribed as not being clearly active or passive and were therefore considered only indirectly as a counterpart of the two variables.

To ensure a basic correlation between the dependent and the independent variables, statistical selection was done in two steps. For all potential variables a univariate regression analysis was calculated. First, all variables whose coefficients exceeded p = 0.25 were eliminated [30]. After that, the multiple linear regression model was calculated backwards stepwise entering all remaining variables. Missing values were excluded casewise as threshold for variable exclusion in the multivariate procedure p≥0.1 was set.

Following the recommendations given by Schendera for identification of outliers, the standardized residuals, the standardized Difference in Fit (DfFit) and Cook-distance were saved [29]. Cases were excluded if standardized residuals exceeded ±3 and if in addition results showed DfFit >2*sqrt(p/n) [sqrt = square root, p = amount of independent variables, n = number of cases] or Cook-distance >1 [29], [31]. The procedure was repeated until no more outliers could be identified.

For testing the significance of the final regression model an ANOVA was carried out. Data were analyzed using SPSS version 19.0.

## Results

84 practices participated with the mean number of therapists in each being 3.4 (SD 2.2). After checking for inclusion and exclusion criteria, results from 792 patients were available for analysis; for catamnesis, data from 391 patients was available (median per practice 4, IQR 2 to 6.5, range 1 to 63). Baseline characteristics are presented in **Table 5**. The mean impairment six months after treatment was 25.3 (SD 22.4).

**Table 5:** Baseline characteristics.

Measure	Overall
	(n = 792)
Mean Age in years (SD)	44.4 (11.4)
Gender female (%)	58.8
Subgroup (%)	
- non-specific low back pain	73.4
- disc/root irritation	17.3
- thoracic spine	9.3
Duration of complaints >1/2 year (%)	56.8
Pain radiation lower extremity (%)	58.4
Mean Impairment prior to treatment (SD)	46.4 (22.4)
Mean pain intensity prior to treatment (SD)	6.0 (2.1)
Mean body mass index in kg/m^2^ (SD)	26.3 (5.3)
Not employed (%)	18.8

**Figure 1:**
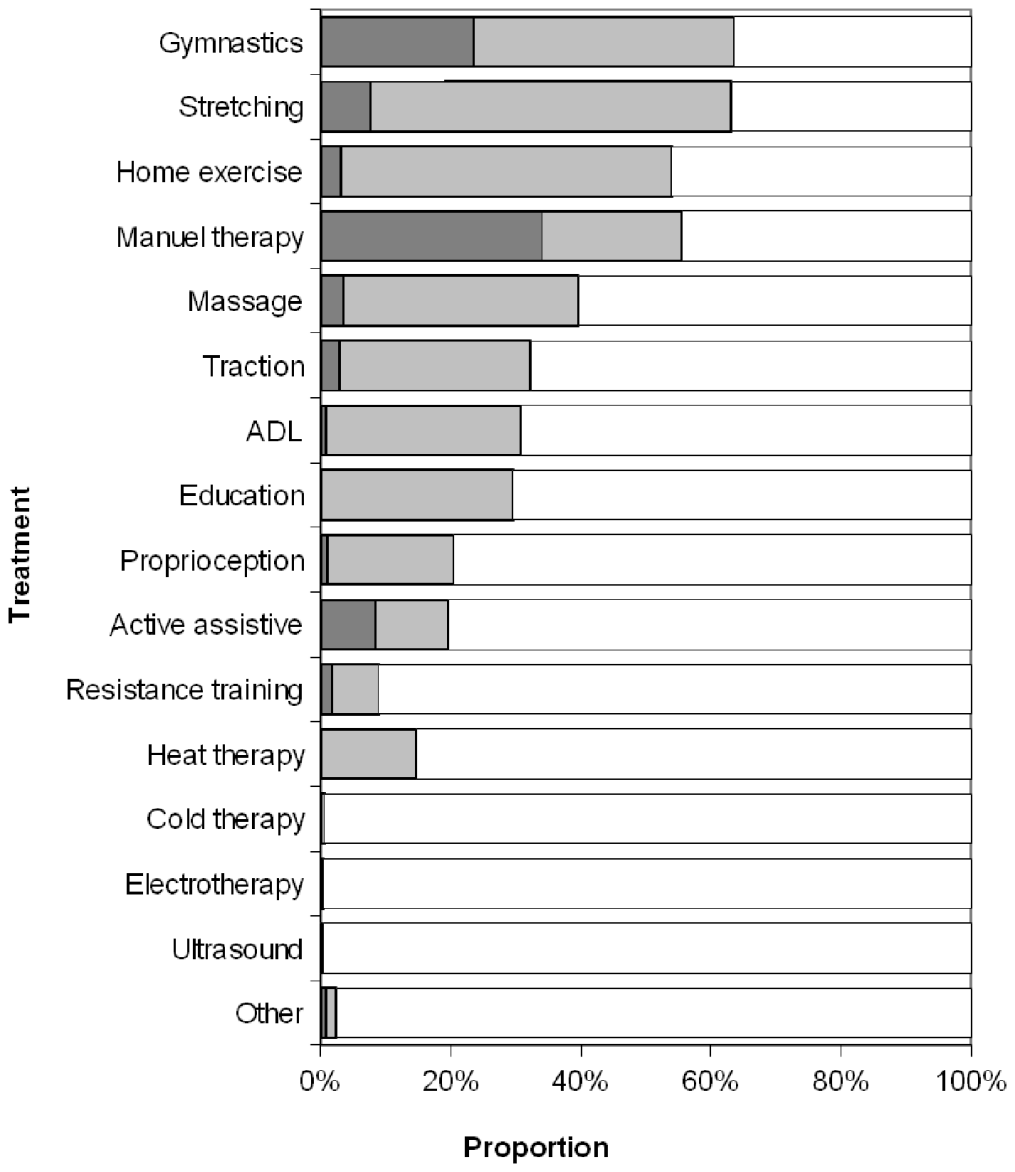
Treatment. Dark grey: primary treatment approach, light grey: secondary. ADL: Activities of daily living.

There were no significant baseline differences between responders and dropouts by age and impairment, but dropouts were more likely to be men (p<0.05). The treatment provided is shown in **Figure 1**.

### Predictors of outcome

Three patients with an extremely high body mass index (>44) were excluded as outliers before final model formulation.

24 variables were adopted in the multivariate regression calculation after univariate analyses because their p-values satisfied the set threshold of 0.25 (see **Table 6**). Differentiated information regarding the unstandardized coefficient B are given in **Table 7**.

**Table 6:** Variables adopted in the multivariate regression analysis.

1. Good self-prognosis on work ability in 2 years	9. Multifocal complaints	17. Subgroup thoracic spine
2. Mental disorder	10. White collar worker	18. Primary therapy passive
3. Impairment prior to treatment	11. Analgesics intake	19. Genitourinary or digestive disease
4. Rheumatoid arthritis	12. Neurological-sensory disease	20. Tumours
5. Mental resources	13. Employed	21. Smoker
6. Duration of complaints ≥½ year	14. Pain radiation lower extremity	22. Diabetes or metabolic disease
7. Pain intensity at t1	15. Physically active	23. Subgroup low back non-specific
8. Age	16. Body mass index	24. Primary therapy active

**Table 7:** Predictors for impairment at 6 Month: Results of univariate Regression Analysis.

Prognostic factor	B	Lower CI	Upper CI
Age	0.47*	0.28	0.66
Gender	0.45	−4.22	5.13
Subgroup non-specific	3.49	−1.48	8.45
Subgroup disc/root irritation	1.12	−4.68	6.92
Subgroup thoracic spine	−10.19*	−17.76	−2.61
Therapy post surgery	−1.93	−26.59	22.72
Impairment prior to treatment	0.34*	0.25	0.44
Pain prior to treatment	2.85*	1.77	3.93
Duration ≥½ year	12.30*	7.96	16.65
Radiation into lower extremity	7.48*	2.97	11.99
Multifocal complaints	14.16*	8.34	19.98
Physically active	−7.43*	−12.05	−2.82
Smoker	4.99	−0.58	10.55
Intake of analgesics	8.84*	4.35	13.32
Body mass index	0.68*	0.23	1.14
Employed	−9.88*	−15.49	−4.28
Mental resources	−7.12*	−9.57	−4.68
Good self-prognosis on work ability in 2 years	−19.69*	−24.53	−14.84
White collar worker	−9.57*	−14.06	−5.07
Rheumatoid arthritis	19.87*	13.47	26.28
Diabetes	9.21	−1.74	20.17
Tumours	11.91	−1.20	25.02
Mental disorder	18.92*	13.64	24.21
Neurological-sensory disease	9.20*	4.18	14.21
Genitourinary or digestive disease	5.49	−0.03	11.02
Primary therapy active	−3.02	−7.58	1.55
Primary therapy passiv	1.94	−2.58	6.45

B = unstandardized Coefficients B, CI: confidence interval,

* P<0.05.

After first regression calculation, two additional outliers were eliminated, since leverage-values exceeded the specified threshold. There were 9 variables identified to have an influence on impairment six months after therapy (**Table 8**). With p>0.05 the body mass index must be regarded as a moderating variable.

**Table 8:** Result of multivariate Regression Analysis.

Independent Variables	Unstandardized Coefficients		Standardized Coefficients	T	p	VIF
	B	SE	Beta			
(Constant)	−3.837	8.066		−0.476	0.635	
Good self-prognosis on work ability	−5.675	2.662	−0.116	−2.132	0.034	1.353
Mental disorder	11.002	2.756	0.202	3.991	0.000	1.181
Impairment prior to treatment	0.205	0.048	0.213	4.251	0.000	1.151
Rheumatoid arthritis	9.368	3.343	0.141	2.802	0.005	1.158
Duration of complaints ≥½ year	8.378	2.213	0.192	3.786	0.000	1.177
Age	0.218	0.097	0.111	2.256	0.025	1.117
White collar worker	−6.233	2.112	−0.141	−2.951	0.003	1.048
Physically active	−4.733	2.126	−0.107	−2.226	0.027	1.064
Body mass index	0.379	0.218	0.086	1.743	0.082	1.123

Dependent variable: Impairment 6 months after therapy.

The sign of the unstandardized regression coefficient B (**Table 8**) shows in which direction the variable influences the prognosis of impairment in daily life half a year after therapy. Metrically scaled predictors “Impairment prior to treatment” and “age” are signed positive. The prognosis thus worsens with higher impairment prior to treatment and/or higher age. Also the dichotomous predictors “mental disorder”, “rheumatoid arthritis” and “duration of complaints ≥½ year” are signed positive. The prognosis thus worsens if the patient suffers from one of the mentioned illnesses and/or has a long history of complaints. The dichotomous predictors “good self-prognosis on work ability in 2 years”, “white collar worker” and “physically active” show a negative sign. If the patient is confident before therapy, is a white collar worker or physically active the prognosis improves.

ANOVA for the final regression model resulted in p<0.001. Our baseline variables predicted 34% of the variance in impairment in daily life 6 months after therapy (adjusted R^2^).

## Discussion

We developed a model for prognosis of disability in back patients half a year after receiving physiotherapy. Outstanding predictors are restrictions in daily life before therapy, mental disorder as secondary diagnosis and duration of complaints; of further relevance are self-prognosed work ability in 2 years, rheumatoid arthritis, age, workplace demands and physical activity. To our knowledge a variable set comparable to ours has not been previously investigated.

At this stage, the comparison of the predictor set as a whole with other studies is not feasible because of the limited number of comparable studies and the diverging sets used. Possibilities for comparison for the variables “age”, “Impairment prior to treatment” and “duration of the complaints” are provided by secondary analysis of two large randomized controlled trials [17], [18]. The strength of the comparison lies within the therapy-specific approach of the trials. In our study, age was a significant factor, as was the case for Underwood et al. However, Bekkering et al. found no such association [17], [18]. Consensus can be found for the significant variable “duration of the complaints” [17], [18]. If the variable “Impairment prior to treatment” is compared with functioning or pain and disability there also can be shown a homogeneous result underlining relevance [17], [18]. A connected predictor was found to be of relevance by another group of researchers, who adopted a follow-up similar to ours. Harms et al. found an episodic pain character to be advantageous [19].

The systematic review by van der Hulst et al. facilitates evaluation for similarities for the variables “physical activity”, “white collar worker” and “self-prognosis” [15]. They also investigated a therapy-specific approach with referral to multidisciplinary rehabilitation or back school. Whether the variable was of importance differed depending on the specific therapy for physical activity. Unlike our results, van der Hulst et al. were not able to describe relevance for the aspect white versus blue collar worker [15]. Reflection on the significant predictors “self-prognosis on work ability” and “mental disorder” is difficult, because many different constructs were investigated in the trials adopted by van der Hulst et al. [15]. An association for the latter may be seen in the Symptom Checklist-90 and the Distress scale, but once more the comparison results in an inconsistency. Findings vary depending on therapy and outcome measure.

The influence of the predictor rheumatoid arthritis can be easily explained by the destructive character of the underlying autoimmune disease [32], [33].

In our study, different subgroups of back pain patients were included. From the relating variables, only the subgroup thoracic spine reached significance and this only in univariate analysis. This result may be partly attributed to the source of the diagnoses, which were taken from the referral-forms and issued by the physician for therapeutic and not for research purposes. According to Thomas et al. pain radiation into the lower extremity may have influence on the persistence of back pain [34]. Also, for our corresponding variable this could have been confirmed only in univariate analysis.

The mean impairment at baseline given for our study is comparable to the results of other research groups [24]. During therapy it was reduced considerably. In a meta-analysis, it was shown for non-specific low back pain patients with chronic complaints that exercise therapy is effective [35]. Therefore, regarding the conducted therapy approaches (**Figure 1**) one may conclude that these had an influence. For passive approaches, results are controversial [36], [37]. However, since some patients had acute complaints the shown reduction could partly be explained by the natural course.

The primary therapy approach was utilized via two variables in our study: Primary therapy approach active or Primary therapy approach passive. Both variables did not reach significance and correspondingly our results do not reinforce the assumption that active approaches show a superior effect. A different allocation of the therapy approaches may have led to different results, moreover in future trials the duration and frequency of therapy may be included as additional factors.

### Implications for practice

For assessment of the need for physiotherapy, the results allow comparison of the patients' therapy odds with the prognosis before treatment starts. Raspe et al. regard this as an essential aspect in needs assessment [14]. In addition, the results add information for compiling more homogeneous cohorts in future experimental studies. Different examinations point to the fact that studies on the efficiency of therapeutic services – seen from a biopsychosocial perspective – are currently carried out with heterogeneous groups of patients. Selected therapy approaches specifically compiled or selected for specified groups of patients could lead to an increase of efficiency in the measures [5], [38], [39].

Three of the four most often used treatment approaches were different types of exercise. This is in accordance with the National Disease Management Guideline for Low Back Pain, in which such approaches are strongly recommended [40]. Manual therapy, the fourth approach is declared as an option. Evidence indicates, that for chronic back pain it may especially be of use in combination with exercise [37], as it was normally conducted by the participating therapists. Particularly the frequent application of traction should be reconsidered. For acute as well as for chronic patients, it is strongly recommended in the guideline not to use it.

Particularly when considering the question about changes of health status after therapy, the developed study design has the advantage that it embodied “usual therapy”, as treatment was not influenced experimentally. The multicentered structure additionally increases the external validity in comparison to a mono-centered design [41]. Moreover, the large sample size can be seen as a strength.

### Study Limitations

For observational studies, the dropout rate for follow-up is a classical challenge [41]. To counteract this, trial-conductance was carefully planned and tested; beyond that the material to be used during the study was developed as user-friendly as possible [42]. In this trial, a response rate of about 50% was obtained; this result is comparable with other prospective studies on musculoskeletal problems [38], [43], [44]. Furthermore, the number of cases made it possible to include all variables, which arose from univariate analyses into final analysis. The higher dropout rate for men may be regarded as a limiting factor for validity.

Multiple linear regression modeling is a complex procedure, which is commonly applied as an iterative process [29], [45]. Correspondingly, in this investigation more than one course of analysis was necessary. Three patients had to be excluded from the analysis before first calculation due to an extremely high body mass index. The values for these patients show a difference of more than three standard deviations from the group average. Thus, the inclusion of them could have led to a distortion of the regression coefficient. Graphical analyses reinforced this hypothesis. Considering recommended thresholds, two additional patients had to be excluded because of their leverage-values [29], [31]. A subsequently performed comparison showed that variables determined as significant before exclusion did not differ from that after exclusion. Correspondingly the coefficients also only differed slightly.

Only exceptionally a regression model leads to a nearby 100% prognosis. However, the level of explained variance with 34% is comparable to that of similarly analysed trials [17], [18].

## Conclusions

In summary, we identified prognostic factors for back patients' impairment in daily life half a year after receiving physiotherapy. Outstanding predictive factors are impairment in daily life before therapy, mental disorder as comorbidity and the duration of the complaints. Our results indicate that prognosis for the individual patient can be estimated and aligned with his or her therapy odds. This may be realistically and simply estimated using a short questionnaire at initial assessment.
